# Isolated splenic lymphangioma presenting as a huge mass causing anemia and abdominal distension in an adult patient: a case report

**DOI:** 10.1186/s13256-018-1664-5

**Published:** 2018-04-16

**Authors:** Boubacar Efared, Gabrielle Atsame-Ebang, Aliou Zabeirou, Nawal Hammas, Khalid Mazaz, Hinde El Fatemi, Laila Chbani

**Affiliations:** 1 Department of Pathology, Hassan II University Hospital, Fez, Morocco; 2 Department of General and Visceral Surgery, Hassan II University Hospital, Fez, Morocco; 3 Faculty of Medicine and Pharmacology, Sidi Mohamed Ben Abdellah University, Fez, Morocco

**Keywords:** Spleen, Lymphangioma, Pathology, Diagnosis

## Abstract

**Background:**

Lymphangiomas are uncommon benign lesions of lymphatic vessels very rarely affecting the spleen. Isolated involvement of the spleen in adult patients is rarely reported.

**Case presentation:**

We report a case of a 40-year-old Arabic woman who presented with a 25-cm abdominal mass, fatigue, and anemia evolving for 6 months. Her physical examination revealed anemic syndrome and an enormous splenomegaly extending beyond the umbilical area. An abdominal computed tomographic scan showed a 25-cm splenic mass with multiple hypodense nodules without enhancement after contrast injection. A surgical total splenectomy was performed. Histopathological analysis led to the diagnosis of cystic splenic lymphangioma. The patient’s postoperative course was uneventful, and she was discharged from the hospital.

**Conclusions:**

Isolated splenic lymphangioma in adult patients is very rare. The preoperative diagnosis is challenging because imaging techniques are not specific. Pathological analysis of the resected specimen is the only effective way to render the definitive diagnosis. Splenic lymphangiomas have a benign course after complete surgical resection.

## Background

Lymphangiomas are benign tumors of lymphatic vessels often located at the neck (75%) and axilla (20%); they are uncommonly found elsewhere [1]. Splenic involvement by lymphangiomas is uncommon, and children rather than adults are mostly affected. Usually, apart from the spleen, other sites are involved, such as the liver, the mediastinum, the lung, the retroperitoneum, or the bone, as part of the so-called lymphangiomatosis syndrome [1, 2]. Isolated splenic lymphangioma is extremely rare; since the first description of a patient with splenic involvement in 1885 by Frink *et al.*, a few cases have been reported in the literature [1–3]. The disease is often suspected incidentally on the basis of imaging or very rarely in symptomatic patients with abdominal pain or distension [2]. The definitive diagnosis is achieved by histological analysis because usually radiologic features of splenic lymphangiomas are not specific and are often misleading [1, 4, 5]. The typical histological aspect of the lesion shows cystic structures filled with eosinophilic, proteinaceous material and lined by flattened endothelial cells [1, 2]. Adequate treatment requires a complete surgical resection [1–3]. In this report, we describe a case of an isolated splenic lymphangioma in a 40-year-old woman presenting with abdominal distension, anemia, and dyspnea.

## Case presentation

A 40-year-old Arabic woman was referred to our hospital for evaluation of a huge splenomegaly causing abdominal pain and shortness of breath. She reported progressive abdominal distension evolving for 6 months, with a recent onset of dyspnea and asthenia. She had consulted a doctor who had prescribed her an abdominal computed tomographic (CT) scan, which revealed a huge splenomegaly of around 25 cm. Her medical history was unremarkable. A physical examination revealed skin pallor and a significant abdominal distension with giant splenomegaly that extended beyond the umbilical area. The patient had no other organomegaly, adenopathy, or skin lesions. Laboratory explorations revealed a microcytic hypochromic anemia with 7.1 g/dl hemoglobin, thrombocytopenia with 98,000 platelets/mm^3^, and a white blood cell count of 6110/mm^3^. The results of other tests were within normal limits. A thoracoabdominal CT scan was obtained, which revealed a splenic mass of 25 cm in larger diameter with regular contours. This mass was made up of many hypodense nodules with variable size and showed no enhancement after contrast injection. The mass was limited to the spleen and pushed the stomach without invasion. No other mass was detected elsewhere. The radiologist suggested the diagnosis of a splenic lymphoma. The result of a bone marrow biopsy was normal at histopathological examination. A surgical total splenectomy was performed.

**Fig. 1:**
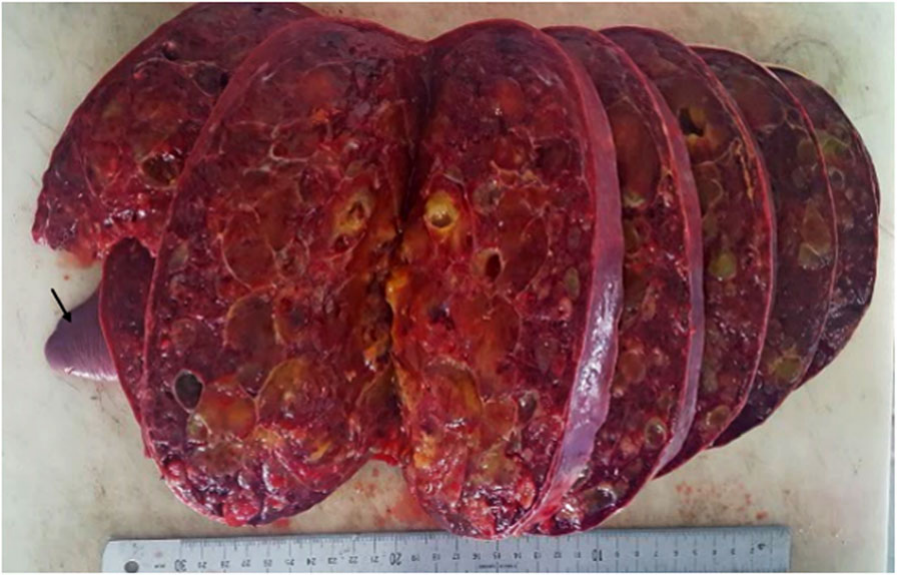
Macroscopic view of the resected specimen shows a huge splenic tumor with a small remaining normal splenic parenchyma (*black arrow*). The cut surface has a honeycombing appearance with multiple cystic structures filled with yellowish material

**Fig. 2:**
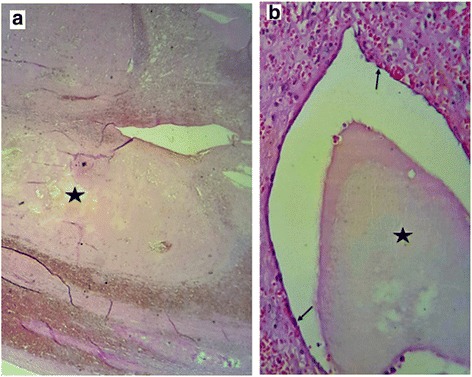
Histological specimen of the splenic lymphangioma shows extensive eosinophilic and proteinaceous material filling cystic spaces (**a**, **b**; *black stars*). These spaces are lined by regular flattened endothelial cells (**b**; *black arrows*)

**Fig. 3:**
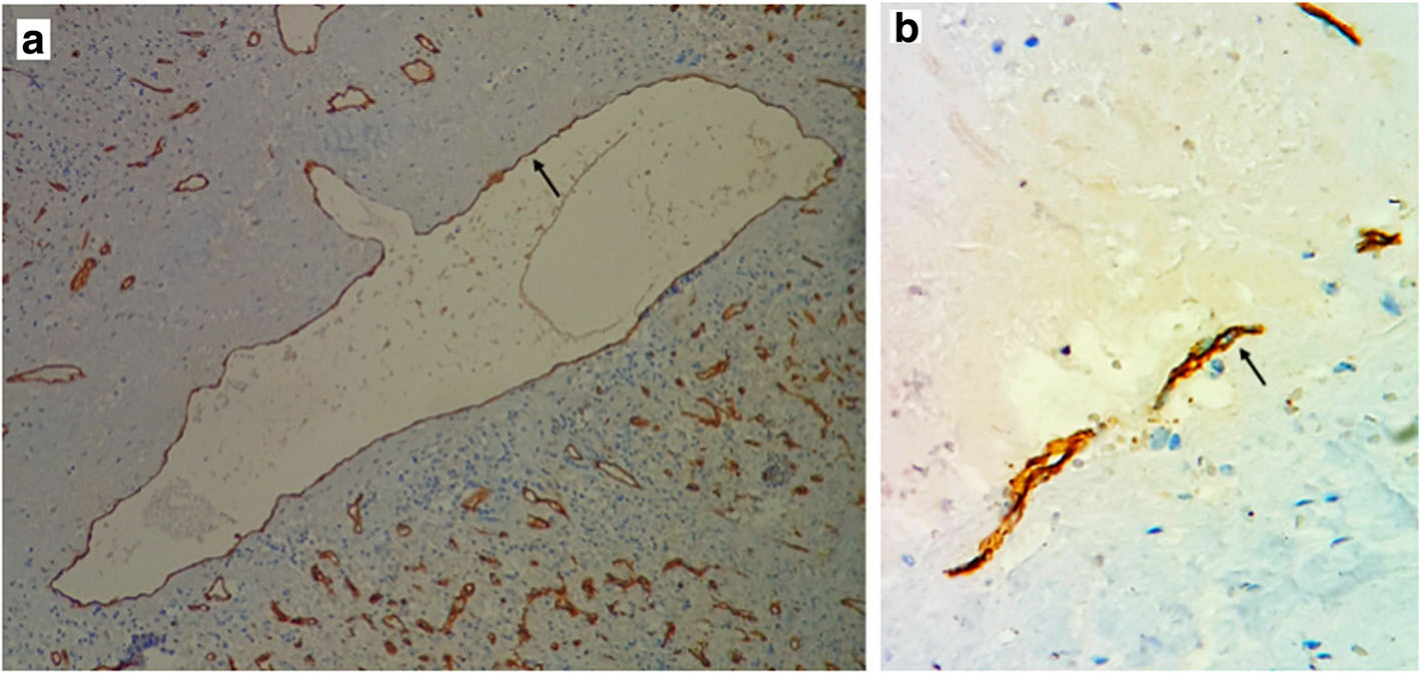
**a** Immunohistochemical image shows a dilated lymphatic vessel lined by CD31-positive endothelial cells (*black arrow*). **b** Higher-magnification view shows flattened endothelial cells with membranous staining by CD31 (*black arrow*)

Macroscopic analysis of the resected spleen showed a giant spleen mass measuring 27 × 23 × 7 cm replacing almost the entire normal parenchyma, with a small residual normal parenchyma (Fig. 1, black arrow). The cut surface of the mass showed multiple cystic structures of variable size, with a “honeycomb” appearance. The cystic structures had a fibrous wall and were filled with a mucoid yellowish substance. Histological examination of hematoxylin-eosin-saffron (HES) stained sections of the tumor showed cystic structures replacing the normal splenic parenchyma and containing amorphous eosinophilic proteinaceous material (Fig. 2, black stars). These cystic cavities had fibrous walls lined by regular flattened small endothelial cells (Fig. 2b, black arrows). In immunohistochemical analysis, these cells stained positive for CD34, CD31 (Fig. 3a, b; black arrows), confirming their endothelial nature. They were negative for epithelial membrane antigen (EMA), pan-cytokeratin (CK AE1/AE3), and calretinin. Thus, the diagnosis was a splenic cystic lymphangioma. The patient recovered well and was discharged from the hospital. Twenty-one months after surgery, she still had no signs of the disease.

## Discussion

We report an unusual presentation of a rare benign splenic tumor. In fact, isolated splenic lymphangioma is a very rare entity; only a few cases have been reported in the literature [1]. Children are mostly affected; 80–90% of lymphangiomas are detected in patients before 2 years of age, and female patients are more commonly affected [2, 6]. More often, other organs are involved as part of the so-called lymphangiomatosis syndrome. Also, splenic lymphangioma can occur in patients with Klippel-Trenaunay syndrome (characterized by varicose veins, bony and soft tissue hypertrophy, cutaneous hemangiomas, and/or malformations of the lymphatic system) [1, 2]. Because lymphangiomas more commonly involve many organs at one time, the diagnostic evaluation should be extended to search for other sites affected by the disease [2]. In our patient, a thoracoabdominal CT scan was performed, and the spleen was the only involved organ.

Splenic lymphangioma is usually an incidental radiological finding because patients are rarely symptomatic. The clinical manifestations are generally due to large tumors with compression of neighboring anatomical structures [2, 3, 6]. Patients with large splenic lymphangiomas often present with left upper quadrant pain, abdominal distension, nausea, or loss of appetite [1]. However, patients with larger lesions may present with complications such as bleeding, consumptive coagulopathy, hypersplenism, or portal hypertension [1, 2, 7]. Our patient had a huge mass of 25 cm. On the basis of her clinical presentation of abdominal distension or dyspnea as a consequence of the tumor size, an anemic syndrome (cutaneous pallor, shortness of breath, low hemoglobin) and thrombocytopenia could have been related to hypersplenism due to the splenic sequestration of red blood cells and platelets, but the patient had no hemorrhagic symptoms.

Usually, splenic lymphangiomas are detected incidentally by radiological techniques if the lesions are small [2, 8]. In these instances, the spleen may be of normal size or may be enlarged, and ultrasonography shows well-defined hypoechoic or anechoic cystic lesions with occasional internal septations or calcifications [1, 2]. On CT scans, lymphangiomas present as subcapsular thin-walled lesions with low attenuation; however, cystic lesions may have intraparenchymal locations if tumors are large [2, 3, 5]. There is no contrast enhancement, just as in our patient. The presence of curvilinear peripheral mural calcifications is very suggestive of the diagnosis of cystic lymphangioma [2]. On T1-weighted magnetic resonance imaging studies, the cystic lesions can appear often hypointense, whereas T2-weighted images show multiloculated hyperintense areas that correspond to the dilated lymphatic spaces [2, 5, 9]. However, all of these radiological aspects are not specific; cases mistaken for other diagnoses such as lymphoma, hemangioma, or hydatid cyst have been reported [2, 4, 9, 10]. In our patient, the radiological features were considered at first as a splenic lymphoma.

In the vast majority of the reported cases, a diagnostic splenectomy was performed, and the pathological examination provided the definitive diagnosis [1, 9–12]. The macroscopic aspects show typically cystic lesions varying in number or in size. Splenic lymphangiomas can present as a unique subcapsular cyst or multiple cystic lesions that can be intraparenchymal or sometimes replace the entire normal splenic parenchyma, as noted in our patient [1–3]. Cystic lesions commonly have thick fibrous walls with a honeycomb appearance if lesions are multiple and confluent [2]. The cysts are filled with an amorphous clear substance. According to the size of the dilated lymphatic vessels, splenic lymphangiomas are classified as capillary, cavernous, or cystic if the abnormal lymphatics have the size of capillaries, the venules, or veins, respectively [2, 3]. In microscopic analysis, the cystic lesions are lined by small flattened endothelial cells, and their lumen is filled by an amorphous eosinophilic proteinaceous material. Cystic walls may be either thin or thick with relatively dense fibrous aspects [1, 2]. There is some controversy regarding the neoplastic or hamartomatous nature of lymphangiomas [2]. The frequent pediatric involvement suggests that lymphangiomas result from abnormal development of lymphatic vessels. Immunohistochemical analysis confirms the endothelial and lymphatic nature of cells lining cystic walls, because they stain positive for CD34, CD31, factor VIII, vascular endothelial growth factor receptor 3, and D2-40 (podoplanin) [1, 3]. Immunohistochemistry is useful to rule out other differential diagnosis when the features of the lesion are not obvious. Hemangiomas, mesothelial cysts, epidermoid cysts, and parasitic cysts (especially due to *Echinococcus granulosus*) are the main histological differential diagnoses [1, 13–16].

Hemangiomas are vascular tumors showing dilated vessels lined by endothelial cells whose lumina are extensively filled with red blood cells, in contrast to amorphous proteinaceous material seen in lymphangiomas. Also, D2-40 immunostaining is negative in endothelial cells of hemangiomas, even though myoepithelial cells are positive for this marker [1]. Mesothelial cysts are lined by cells that stain positive for markers such as calretinin or WT-1 and negative for endothelial markers, whereas epidermoid cysts are lined by epithelial cells positive for cytokeratins [13, 14]. The hydatid cyst consists of three layers (innermost germinal layer, intermediate laminated membrane, and outer fibrous layer) and contains protoscolices [1–3, 15]. Our patient had typical histological features of splenic lymphangioma, and we used immunohistochemical staining by CK AE1/AE3 and EMA to exclude the diagnosis of epidermoid cyst and calretinin to rule out the diagnosis of a mesothelial cyst.

Splenic lymphangiomas have a benign course, even though rare cases with sarcomatous transformation have been reported [1]. Complete surgical resection is the treatment of choice because other therapeutic options, such as aspiration, drainage, or radiation, are not efficient [1, 4, 6]. Total splenectomy is an adequate therapeutic option, especially for large lesions, in order to prevent complications such as splenic rupture, infection, hemorrhage, intestinal obstruction, or recurrence [2, 9, 11].

## Conclusions

Lymphangiomas are rare benign tumors that often present with multiorgan involvement in children. The occurrence of lymphangiomas as isolated lesions of the spleen in adult patients is extremely uncommon. The preoperative diagnosis in this context is misleading, and splenectomy for therapeutic and diagnostic purposes is the only appropriate management.

## Abbreviations

CK: Cytokeratin
CT: Computed tomography
EMA: Epithelial membrane antigen
